# Challenging assumptions about “moving online” in response to COVID-19, and some practical advice

**DOI:** 10.15694/mep.2020.000083.1

**Published:** 2020-04-30

**Authors:** Tim Fawns, Derek Jones, Gillian Aitken

**Affiliations:** 1 University of Edinburgh

**Keywords:** COVID-19, online learning, open learning, distance learning, e-learning, advice

## Abstract

This article was migrated. The article was marked as recommended.

COVID-19 is forcing many Universities to close for some time and programmes for medical, allied health, and nursing students are moving online. Based on our extensive collective experience teaching a variety of health professionals in clinical and academic settings, and of online learning, we want to question assumptions that seem apparent to us in some of the discourse around “moving teaching online”. We write from the perspective of a team delivering a postgraduate programme for professionals working full time (in most cases) from around the world and different professions; however, we believe the issues raised are applicable to online education in general. There is a practical purpose to confronting these assumptions. It is our aim to help those delivering health professions education avoid some pitfalls that we think would result in poor quality experiences for all concerned. We do not believe that online education is inherently poorer nor, indeed, fundamentally distinct from on-campus education. However, there are certainly important considerations for learning to teach online, and we attempt to highlight some of these. We also provide some advice to those new to teaching online based on our experience, research, and the literature.

## Introduction

As universities face temporary closure due to COVID-19, many medical, allied health, and nursing schools are moving their undergraduate programmes (or at least significant elements of them) online. Based on our extensive collective experience teaching health professionals in clinical and academic settings, of online learning, and of delivering a highly rated successful online MSc in Clinical Education, we want to question some assumptions that seem apparent to us in some of the discourse around “moving teaching online”. We write from the perspective of a team delivering a postgraduate programme for professionals working full time (in most cases) from around the world and different professions; however, we believe the issues raised are applicable to online education in general. The assumptions we want to address are as follows.

• Assumption 1: What is being moved online is a curriculum.
• Assumption 2: The challenges of moving online are technical and skills based.
• Assumption 3: Changing from oncampus to online can be done smoothly, without significant additional time, support, and faculty development.
• Assumption 4: The principles of teaching online are somehow different from on campus.

There is a practical purpose to confronting these assumptions. To manage this process of reconfiguring teaching and learning, educators need to have a realistic and pedagogically-informed understanding of what they are dealing with. It is our aim to help those delivering health professions education avoid some pitfalls that we think would result in poor quality experiences for all concerned. As we have expressed before, we do not believe that online education is inherently poorer ( Fawns *et al*., 2019), nor, indeed, fundamentally distinct from on-campus education ( Fawns, 2019). However, there are certainly important considerations for learning to teach online, and we attempt to highlight some of these below. We also provide some advice to those new to teaching online based on our experience, research, and the literature.

## Assumptions About “Moving Online”

### Assumption 1: Teaching, or a course, can be “moved online”

It is understandable that, in the face of sudden campus closures, medical schools and teachers would want to retain as much of their existing educational provision as possible. There is already significant upheaval, and little time for wholesale programme redesign and development. However, it is risky to assume that maintaining current teaching strategies and uploading existing content to an online platform (perhaps recording it or translating it into a web-based text first) will produce the same kind of educational experience as previously. For one thing, education is only partly about content, and for many teachers the content is inseparable from the person delivering it and their interaction with students ( Shulman, 1987). Content gives us something to work with, but it is often the social activity, the relationship-building, the problem-solving, the dialogue and generation of ideas, and the students’ own discovery of other content that has not been pre-defined by the teacher, where much of the value of education is to be found ( Wright, 2011; Slavich and Zimbardo, 2012).

The spaces in which these activities take place shape the ways in which students and teachers communicate and develop particular practices of learning and thinking. The interaction moves online, but the course does not ‘move’. To imagine that it is the course that moves is to engage in *reification*, that is, to treat it as a ‘thing’. What happens is in fact a transformation of something that is both social and material, in ways that are not under the control of the teacher. The teacher must still design the course, and it is composed of some things (materials) such as videos and journal articles; but online education highlights the extent to which this design is just a starting point for dynamic activities that can only be orchestrated and not determined ( Fawns, 2019). It is not like a recording but more like a live orchestra, where music is made by the coming together of listeners, the composer, written score, musicians, venue, conductor, musicians, and specific instruments. A ‘course’ cannot be moved online, because it is not a simple static, portable, thing. It is made up of the emergent and interdependent activities of teachers, students, administrators, learning technologists, technologies, and social and material, physical and digital environments ( Fawns *et al*., 2020). Further, the learning does not stay online, but spills out beyond the course and into the home and professional environments of students and, potentially, their peers and colleagues ( Aitken, 2020; Aitken *et al*., 2019).

### Assumption 2: The challenges of moving online are technical and skills based

Of course, in day to day life, educators are often required to deviate from their lesson plans to accommodate unexpected scenarios as they arise. Fire alarms go off, the projector breaks down, a lecture is cancelled, and content is not covered. However, moving online is not just about adding new steps to some already fancy footwork. For some teachers it can provoke the kind of challenge that might be experienced by a classically trained ballet dancer moving to contemporary dance. Traditional notions of rhythm, movement and form become exposed and may require a shift in mindset. The sudden need to rethink what we do brings such issues to the surface, but they are not new. These were always important issues in education. Our traditional methods allowed us to take them for granted in many cases, particularly where insufficient time was allocated, not just to teaching, but to learning how to teach. Whilst the institutional structures may be oriented in terms of technical and skills-based challenges, for the teacher the challenge can also be much deeper.

Take the large-scale lecture. We often hear of teachers recycling old material because they do not have the time to revise it. There are criticisms of poor PowerPoint slides and praise for good ones (or suggestions to use alternatives, such as Prezi) or for the engaging performance of the speaker. However, to judge a lecture in relation to its content, or even the presentation skills of the lecturer, is to ignore core elements of its potential value. A lecture contains the possibility to be more than (to quote an often-used adage) the transmission of the notes of the lecturer to the notes of the student without passing through the mind of either. Face to face or online, it can be motivational, inspirational, and organisational (helping students understand what they should do after the lecture, or how other aspects of the curriculum fit together). It can encourage students to gather together, before and afterwards, and discuss relevant issues. It can allow pastoral encounters or, at least, for teachers to see that their students are well enough to be present. For the lecturer who manages to do all of these things, recreating these functions requires more than recording the lecture and putting it online.

Worries have been expressed by educators, students and parents of undergraduates about how it is possible that programmes can have the same outcomes online as on-campus, or worries that online is isolating, or that students will be left to fend for themselves ( Fazackerley, 2020; Thompson, 2017). These are legitimate concerns, but we can, and should, use this situation to think about the shortcomings that may already be present in on-campus education (e.g. teachers working in silos and being unaware of the wider curriculum, or, indeed, students being left to fend for themselves). We can use these experiences to think about how we might do better when things settle back into a routine. For example, while electronic attendance registers for face to face teaching and learning analytics for VLEs have been used to police attendance and engagement, our heightened sensitivity to the wellbeing of students might lead us to use these devices only as pastoral supports by identifying those who have not logged in to the central point of contact (e.g. the Virtual Learning Environment). We might shift from a policing function to a pastoral one, by using these measures to check that students are coping, rather than to judge or admonish them. This would acknowledge that learners (particularly in our postgraduate context) make choices about how they engage. The students on our postgraduate programme are already experienced (if not necessarily efficient), driven, learners. Most know what they need to do in terms of working through materials and submitting a good enough assignment on time. For, typically younger, undergraduate students, this may not be the case. We should not forget that students, as well as staff, may need support to adjust to learning online, which is about more than how to post on a discussion board or log into a video-conference tutorial.

### Assumption 3: Moving teaching online can be done smoothly, without significant additional time, support, and faculty development

We cannot expect teachers to radically revise their designs, content, and overall pedagogical perspectives as they rush (or are pushed) online. Nonetheless, they cannot simply continue as normal. The primary reason for this may be the significant additional work of dealing with the uncertainty and the concerns of the students. There is no way to avoid upheaval, and the extra strain of this upheaval should be recognised in changes to the workstreams of teachers, administrators and others who support education. It is not just the change in ways of working, but the need to learn and test out these new ways. All of this at a time when (in the UK, at least) academics have been taking industrial action over pensions, workloads and increased use of short-term contracts.

An additional worry is that educational leadership might succumb to the advances of educational technology companies that are seizing the opportunity to sell ‘oven ready’ technological solutions. This is not the time to try out new and unfamiliar platforms, and students need to be protected from rushed decisions that may not take sufficient account of their privacy, or data ownership ( Williamson, 2016), or protect them from companies seeking to leverage their intellectual work in the same way social media extracts value from surfers’ online activity. Further, many technological platforms offer services that employ coercive behaviourist principles ( Knox *et al*., 2019) that utilise reductive and oppressive metrics of engagement ( Fawns *et al*., 2020). As we have argued ( Fawns *et al*., 2019), there are no shortcuts to producing the trusting relationships between students, teachers and institutions that underpin quality online education.

### Assumption 4: The principles of teaching online are somehow different

Our critique of this final assumption should give us hope. While there are important differences between teaching in a physical classroom and teaching online, we believe that they are not fundamentally different with respect to the principles of good quality education. In each setting, teachers must come to understand their hopes for their students (which may be negotiated with them) and use these as the basis for the design of tasks and environments that promote learning. In each setting, however, the teacher does not have full control over what the students do or how the environment is shaped and configured over time. Students will always subvert the designs of teachers, just as teachers will subvert the policies, systems and technologies of their institution ( Fawns, 2019; Fawns and O’Shea, 2019). One advantage of the present crisis is that this subversion is now legitimated in the context of doing whatever it takes to keep business going.

That is not a reason for teachers to relinquish responsibility. Instead, their responsibility lies with engaging with students to understand what it is they are doing, how they are learning, and where the teacher can help them to refine their practices, connect with peers, find appropriate learning resources, etc. In this case of responding to large-scale change, teachers need to keep in touch with students to see how their approach may need to change. There needs to be a Plan B if Plan A does not work out. Even if Plan A works, there should be discussion as to how to proceed once the threat is over: what is the plan for supporting students once the campus reopens? How can teachers make sure they have learned from this experience as they re-join the physical campus and maintain positive changes that may have been made?

## Practical Advice

The Hungarian Marxist philosopher Georg Lukács (1971) berated some fellow academics for residing in the Grand Hotel at the edge of the abyss - living in comfort whilst analysing in a detached way the crisis around them and doing little to intervene. We would not want to leave ourselves open to this charge, so here is our advice to those caught up in responding at speed and scale to the need to move teaching online. Our advice is primarily for teachers - changing the behaviour of institutions may take a little longer.

1. Do not try to do the same thing online as you do face-to-face. Instead, think about what you are trying to achieve, what tools and resources are available, and how you can get students to engage with them as simply as possible.
2. At the same time, try to keep things simple. Where possible, use the tools that are already familiar to you and students.
3. Go low-tech where possible and allow alternative ways of working when not connected. Do not assume all students will have stable connectivity, access to good quality or large screen devices, or even a quiet environment in which to work. Many of our students in low income countries (and some remote and rural areas of the UK) have unreliable wi-fi. We have produced short workbooks that support the completion of an assignment and can be downloaded and printed. These are highly valued by many students who cannot always be connected via the Internet.
4. Be flexible, because students will struggle for various reasons that may not be predictable. Think about how to manage responses to student queries even when they arrive out of hours. Remember pastoral needs. Of course, you also need to think of your own health and well-being. Our team is used to working flexibly as our students span time zones: our working day can be their evening or weekend (or vice versa). Whatever your normal work pattern, make sure students know what this is in the online space. Online teaching can take place anywhere, anytime - but it doesn’t have to.
5. Some tutors new to teaching online worry that their competence may be called into question if they appear unable to work the technology. Our experience is that if you promote a culture of working together and problem solving (rather than “this is the way it is going to work, and you will comply”), learners are generally very forgiving.
6. Teaching involves a degree of emotional labour and relationship building. As health professionals, we are trained to treat patients as people first, and most health professionals working in education apply the same ethic to students. For this reason, at first sight, online teaching might seem to be a barrier to the very thing we enjoy about teaching. However, it doesn’t have to be this way. In one of our papers ( Fawns *et al*., 2019) we cite the musician Bjork who said that “If electronic music has no, soul it is because nobody put it there.” The same applies to online teaching. The level of engagement that is possible came as a surprise to participants in Aitken & Loads’ (2019) paper describing the experiences of educators new to online teaching.
7. When using discussion boards, avoid questions where all that can be said has been said after the first half dozen responses. Asking participants to share experiences pertinent to the topic under discussion gives everyone an opportunity to say something. Moreover, unlike the classroom situation, students have the opportunity to allow their thoughts to percolate before committing to a response. For this reason, we often find that people who may be less active in live discussions (face to face or online) have more to say in asynchronous discussion boards.
8. Many of the risks associated with email communication apply to written online communication. Consider using video or audio clips to provide feedback or give instructions (avoiding reading from a script - it may sound odd at first, but who cares if you pause or stumble over a word). When crafting discussion board postings, remember that you are modelling to your students how you want them to communicate with each other in terms of tone and content. In most cases, you want to be courteous but informal. If you want to generate a conversation rather than a question and answer activity, this is not the place to be fussy about grammar and referencing (though it is a great place to share resources). Allow people to go off topic, you never know where it might lead.
9. Resist the temptation to overload your VLE with content. A five-page reading list is no more helpful online than it is in face to face teaching. Use content to generate discussion and engagement - less is more.
10. A big advantage of online learning is that it gives more control to the learner, so focus less on synchronous events (live sessions) and more on asynchronous activities (wikis, discussion boards, etc.). Group projects can be really good but challenging to organise. In the present circumstances, students may have less control over space to work, have Wi-Fi download limits or low bandwidth, or be spending time queuing to get into the local supermarket.
11. If you have neglected your relationships with your colleagues in IT/Information Services, now is the time to be building those bridges and showing appreciation for the unsung work they do behind the scenes. Also, track down your colleagues who have experience of online teaching, they may appreciate a fresh pair of eyes on their work as well as being able to offer you some advice.

## Conclusion

Our experience and research into online teaching leads us to conclude that, despite its challenges, it can be enormously enjoyable and effective. The current situation presents us with a strong reason to engage with it and (dare we say) bend a few ‘rules’ and challenge taken for granted assumptions.

## Take Home Messages

• What is being moved online is not a curriculum, because a curriculum is not a ‘thing’ that can be ‘moved’.
• The challenge of moving online is about more than technical issues and skills.
• Changing from on-campus to effective online teaching requires time, support, and faculty development.
• The principles of learning and teaching online are not different from learning and teaching on campus.
• Work with your learners and consider the challenges they might face moving online.

## Notes On Contributors

**Tim Fawns** is the Deputy Programme Director of the MSc Clincial Education programme at the University of Edinburgh.

ORCID iD: https://orcid.org/0000-0001-5014-2662.

**Derek Jones** is the Programme Director of the PhD Clinical Education programme at the University of Edinburgh.

ORCID iD: https://orcid.org/0000-0003-2197-7657.

**Gill Aitken** is the Programme Director of the MSc Clinical Education programme at the University of Edinburgh.

ORCID iD: https://orcid.org/0000-0002-5492-1943.

## Declarations

The author has declared that there are no conflicts of interest.

## Ethics Statement

Ethical permission was not required as our institution does not require approval for discussions of practical tips and/or guidance.

## External Funding

This article has not had any External Funding
