# Supplementary material for: Challenging assumptions about “moving online” in response to COVID-19, and some practical advice
Source: MedEdPublish (2016). 2020 Apr 30;9:83. [Version 1] doi: 10.15694/mep.2020.000083.1 (PMC10697494; doi:10.15694/mep.2020.000083.1)
Supplement: Peer review history

# Reviewer report: Reviewer response for version 1

**DOI:** 10.21956/mep.20065.r30808

**Published:** 2020-04-30

**Authors:** PATRICIA CURY

**Affiliations:** 1 FACERES

This review has been migrated. The reviewer awarded 5 stars out of 5 This is a very interesting paper that every faculty team should read. Every now and then we forget that this is happening all over medical schools and these thoughts can be observed or used by all. One think that I would like to add is our strategy to integrate the student with our moment is to always is possible, we use COVID-19 topics (for example, with elderly people problems, ethical problems about patients and medical choices, antiviral drugs , etc.…).

Reviewer Expertise:

NA

No decision status is available

# Reviewer report: Reviewer response for version 1

**DOI:** 10.21956/mep.20065.r30810

**Published:** 2020-04-30

**Authors:** James Gray

**Affiliations:** 1 University of Sheffield

This review has been migrated. The reviewer awarded 4 stars out of 5 An interesting article looking at the issue in a slightly more philosophical way and delivering a degree of reassurance for all of us working in these challenging times. There are some helpful practical suggestions here which we can take back as well as some related to our own self management in these times. Certainly helpful when thinkign how to support other teachers in moving to online technologies. An interesting read

Reviewer Expertise:

NA

No decision status is available

# Reviewer report: Reviewer response for version 1

**DOI:** 10.21956/mep.20065.r30811

**Published:** 2020-04-30

**Authors:** Judy McKimm

**Affiliations:** 1 Swansea University

This review has been migrated. The reviewer awarded 5 stars out of 5 Many thanks for writing this article reflecting your own experience as well as drawing from the literature. I think that the assumptions you mention are very central to challenge, and perhaps I would move the fourth to be the first, going back to basics re both how you might achieve the formal LOs and the learning environment is often where I start. Plus, what doesn't need to be done, and how will this work for all learners, especially in very low resource settings (which might not be in low resource countries). I strongly recommend this article, not just right now as we are all dealing with the impact of the pandemic, but as we move forward, so that lessons we are learning now can be embedded into our future practice.

Reviewer Expertise:

NA

No decision status is available

# Reviewer report: Reviewer response for version 1

**DOI:** 10.21956/mep.20065.r30809

**Published:** 2020-04-30

**Authors:** Trevor Gibbs

**Affiliations:** 1 AMEE

This review has been migrated. The reviewer awarded 5 stars out of 5 In difficult times , it is important to see all sides to the problem- this paper I feel does just that. I really enjoyed reading this paper and gained a lot of personal learning from it, finding myself, at the same time, fully agreeing with the authors. It was a very refreshing paper, moving away from the assumptive givens that we are all now expecting. I particularly valued the push towards the more humanistic influences on teaching and learning- if education has no sole, that is because no one put it there!I strongly recommend this paper to all educationalists, if only to remove some of those educational pressures that are presently on our shoulders.

Reviewer Expertise:

NA

No decision status is available
